# Iron deficiency affects nitrogen metabolism in cucumber (*Cucumis sativus* L.) plants

**DOI:** 10.1186/1471-2229-12-189

**Published:** 2012-10-11

**Authors:** Andrea Borlotti, Gianpiero Vigani, Graziano Zocchi

**Affiliations:** 1 Dipartimento di Scienze Agrarie e Ambientali - Produzione, Territorio, Agroenergia, Università degli Studi di Milano, Via Celoria 2, I-20133, Milano, Italy

**Keywords:** C/N metabolism, *Cucumis sativus* L., Fe deficiency, GS/GOGAT cycle, Isocitrate dehydrogenase, Nitrate reductase

## Abstract

**Background:**

Nitrogen is a principal limiting nutrient in plant growth and development. Among factors that may limit NO_3_^-^ assimilation, Fe potentially plays a crucial role being a metal cofactor of enzymes of the reductive assimilatory pathway. Very few information is available about the changes of nitrogen metabolism occurring under Fe deficiency in Strategy I plants. The aim of this work was to study how cucumber (*Cucumis sativus* L.) plants modify their nitrogen metabolism when grown under iron deficiency.

**Results:**

The activity of enzymes involved in the reductive assimilation of nitrate and the reactions that produce the substrates for the ammonium assimilation both at root and at leaf levels in Fe-deficient cucumber plants were investigated. Under Fe deficiency, only nitrate reductase (EC 1.7.1.1) activity decreased both at the root and leaf level, whilst for glutamine synthetase (EC 6.3.1.2) and glutamate synthase (EC 1.4.1.14) an increase was found. Accordingly, the transcript analysis for these enzymes showed the same behaviour except for root nitrate reductase which increased. Furthermore, it was found that amino acid concentration greatly decreased in Fe-deficient roots, whilst it increased in the corresponding leaves. Moreover, amino acids increased in the xylem sap of Fe-deficient plants.

**Conclusions:**

The data obtained in this work provided new insights on the responses of plants to Fe deficiency, suggesting that this nutritional disorder differentially affected N metabolism in root and in leaf. Indeed under Fe deficiency, roots respond more efficiently, sustaining the whole plant by furnishing metabolites (i.e. *aa*, organic acids) to the leaves.

## Background

Nitrogen (N) is one of the most important inorganic nutrient in plants because it is a major constituent of proteins, nucleotides, as well as chlorophyll and numerous other metabolites and cellular components [1]. Furthermore, nitrate (NO_3_^-^) is the most abundant anionic nutrient in aerobic soil and is taken up from the soil solution by transport across the plasma membrane of epidermal and cortical cells of the root, involving an inducible high-affinity transport system [2,3]. Nitrogen is often a limiting factor for plant growth and development. There is keen interest and considerable potential agronomic benefit in the understanding of the mechanisms that determine N use efficiency and in identifying targets for improvement.

Among the factors which may limit NO_3_^-^ assimilation, iron (Fe) plays a crucial role, being a metal cofactor of enzymes of the reductive assimilatory pathway [nitrate reductase (NR), nitrite reductase (NiR) and glutamate synthase (GOGAT), all requiring Fe as Fe-heme group or Fe-S cluster] [1]. It has been shown that Fe deficiency induces various responses at the root level that increase the availability of the ion in the rhizosphere. Strategy I plants (dicotyledonous and non-graminaceous monocots) are able to respond to a shortage of Fe in the soil by increasing: (i) the Fe reduction capacity of root tissues [Fe^3+^-chelate reductase (EC 1.16.1.7] (FC-R), (ii) the acidification of the rhizosphere to increase Fe solubility [P-type H^+^-ATPase (EC 3.6.3.6)] and (iii) uptake activity in rhizodermal root cells (Iron Regulated Transporter 1 [IRT1]) [4-6]. The Fe uptake mechanism is tightly regulated by a complex system involving several basic helix-loop-helix (bHLH) transcriptional factors, and of these FIT/FER (FER-Like Iron deficiency-induced Transcription factor) and PYE (POPEYE) play a central role [7] and references therein.

However, there is fragmentary information about the change in N metabolism that occurs under Fe deficiency [8-11]. From microarray data some information is available concerning the Fe-deficient-dependent expression of genes related to N metabolism in *Arabidopsis*[12-14]. The effect of Fe deficiency on NO_3_^-^ uptake has been documented. In fact, a decrease in NO_3_^-^ uptake was observed in Fe-deficient cucumber plants [15]. Wang et al. [16] found that under Fe deficiency tomato roots induce the expression of nitrate transporter LeNRT1.2, but not LeNRT2.1. Potentially, both NO_3_^-^ assimilation and Fe acquisition could compete for reducing equivalents; thus a limitation in NO_3_^-^ uptake could favour the reduction-based mechanism of Fe uptake [17] or, alternatively, since NO_3_^-^ reduction is carried out by Fe-containing enzymes (i.e. NR and NiR), Fe deficiency could alter the cytosolic NO_3_^-^ concentration leading to a restriction of its uptake mechanism(s).

At the metabolic level Fe deficiency induces several changes mainly concerning carbon (C) metabolism [18-20]. C and N metabolisms are strongly interrelated. Numerous studies have shown that C and N metabolites are monitored by the cell and act in concert to orchestrate gene expression, thus determining transcript profiles that are appropriate to nutritional and metabolic status [21-23]. C/N interaction takes place within a context of energy use and production involving cooperation between different subcellular compartments [24-30]. NO_3_^-^ assimilation requires reductants to be supplied to both NR and NiR. However, if NR activity is directly limited by the level of NADH in plants remains an open question [31]. Kaiser et al. [32,33] reported that in spinach leaves, the low photosynthetic activity may limit NR activity through decreased reductant availability, as well as through post-translational inactivation. NO_3_^-^ reduction and mitochondrial electron transport may compete each other for reductants under some conditions. In addition to NO_3_^-^ reduction, a second process that may be subject to redox modulation in the C/N interaction is the formation of organic acids. Organic compounds such as 2-oxoglutarate (2-OG) act as carbon skeleton acceptors of amino groups in the biosynthesis of amino acids and their production requires oxidation through the respiratory pathways [25,34-36]. Our understanding of the significance of mitochondrial metabolism in the production of carbon skeletons for ammonia assimilation has advanced considerably in recent years [37]. As amino acid synthesis involves nitrate reduction occurring alongside carbon oxidation, redox status may be an important factor in the integration of the two processes. Under Fe deficiency the mitochondrial respiratory chain is impaired [38] and the C/N metabolism undergoes strong modification. The aim of this paper is to provide new insights on N metabolism under Fe deficiency. The data collected in this research show that Fe deficiency has a differential effect on N metabolism in roots and leaves, with particular adaptive mechanisms to this nutritional constraint acting at the whole plant level.

## Results

**Figure 1:**
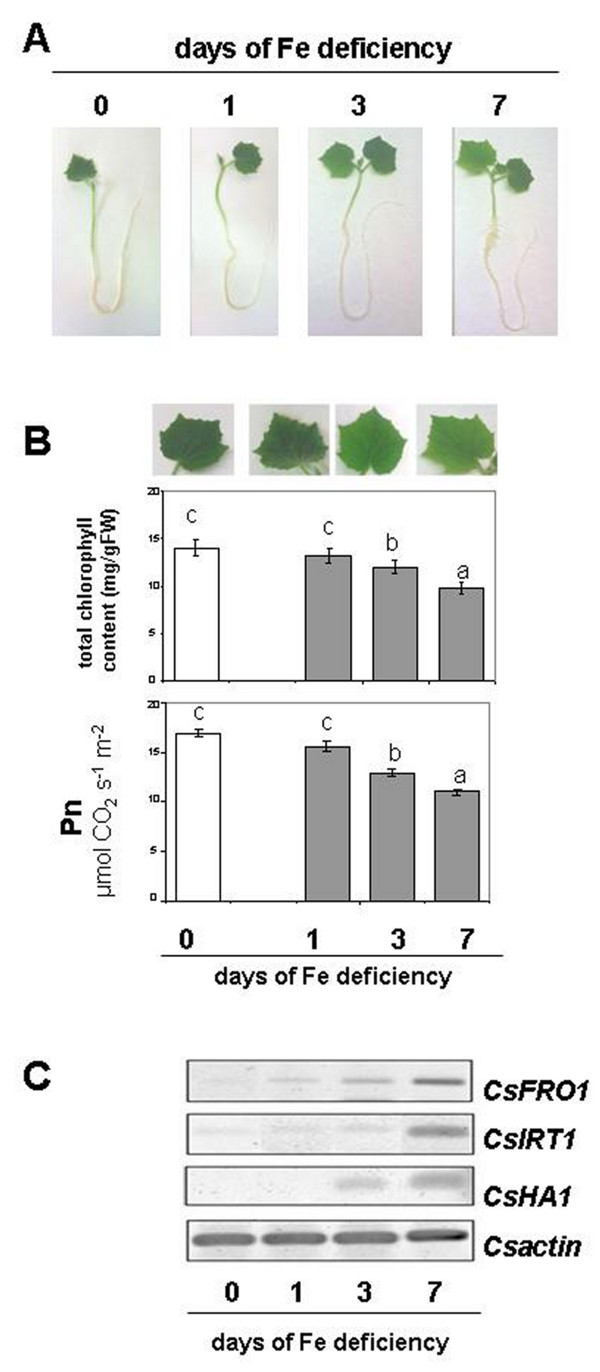
**(A) Effect of Fe-deficient treatment on plant growth, (B) chlorophyll concentration and photosynthesis, (C) RT-PCR analysis of the expression of Strategy I genes (*CsFRO1, CsHA1 *and *CsIRT1*) in roots.** Sampling was done at 0, 1, 3, 7 d after Fe withdraw. Data are means ± SE (n = 4). In the case of significant difference (P<0.05) values with different letters are statistically different.

In this work the change in the N metabolism in response to Fe deficiency has been studied at root and leaf levels. We have re-examined the responses to Fe starvation in cucumber plants previously grown for 7 d in the presence of Fe and, after this period, deprived of the micronutrient. Figure 1 shows the difference among plants grown under these conditions (Figure 1A), the chlorophyll concentration, the net photosynthesis rate (Pn) and the expression of Strategy I genes. Under these conditions leaves show visible symptom of chlorosis with a concomitant decrease in both the chlorophyll content and in the Pn already after 3 d of starvation (Figure 1B). In order to guarantee that Fe-deficient plants developed clear responses in agreement with Fe-deficient condition, a preliminary time course experiment on the expression of the Strategy I genes was performed at the root level (Figure 1C). The expression of *CsFRO1*, *CsHA1* and *CsIRT1* increased in response to the lack of Fe in agreement with data reported in the literature [39,40].

### Effect of Fe deficiency on NR activity and gene expression

**Figure 2:**
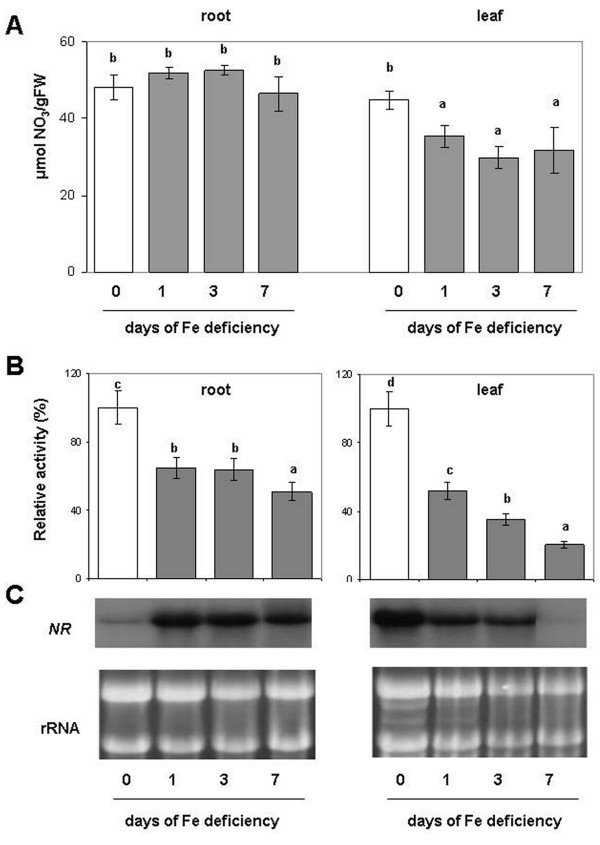
**(A) Nitrate concentration, (B) enzymatic activity and (C) Northern Blot analyses of NR.** Assay and northern blot were performed on root and leaf of plant during the progression of Fe deficiency treatment. Sampling was performed at 0, 1, 3, 7 days after Fe withdraw. The activities are expressed as percentage (%) of the relative activity of the Fe-deficient samples (columns 1, 3, 7 d) compared to Fe sufficient plants (column 0 d). Activity of control (0 d) was 5.6 and 9.8 nmol NADH mg^-1^ prot min^-1^, respectively for root and leaf (complete activity data are reported in Additional file 1). Data are means ± SE (n = 4). In the case of significant difference (P<0.05) values with different letters are statistically different.

Figure 2A shows the concentration of NO_3_^-^ both in roots and in leaves. No significant differences among treatments were found for roots, while a decrease (−35%) was found in the leaves in all the days assayed after induction of Fe deficiency. The NR activity, which is the first enzyme in the NO_3_^-^ assimilatory pathway, decreased both at root and at leaf level during the progression of the Fe deficiency-induced condition (1, 3, 7 d of -Fe). However, in Fe-deficient leaves, NR activity decrease was much more evident as compared to the root reaching −80% after 7 d, suggesting that Fe deficiency affected more the NR activity in the leaves than in the roots (Figure 2B and Additional file 1). On the contrary, under Fe deficiency, the NR transcript increased in roots, while in leaves its expression was strongly decreased, as revealed by northern blot analysis (Figure 2C). These changes in the transcript levels were already evident 1 d after Fe removal.

### Effect of Fe deficiency on ICDH activity and gene expression

**Figure 3:**
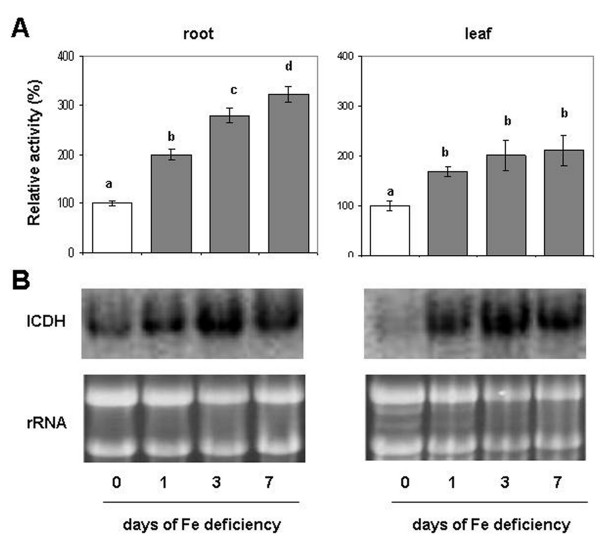
**(A) Enzymatic assay and (B) Northern Blot analysis of cytosolic ICDH in roots and leaves of cucumber plants during the progression of Fe deficiency treatment.** Sampling was performed at 0, 1, 3, 7 days after Fe withdraw. ICDH control activity was 92 and 98 nmol NADPH mg^-1^ prot min^-1^, respectively for root and leaf (complete activity data are reported in Additional file 1). The meaning of the columns and the measurement units of enzymatic activities are as reported in Figure 2. Data are means ± SE (n = 4). In the case of significant difference (P<0.05) values with different letters are statistically different.

Figure 3A shows the time course of the ICDH (cytosolic isoform) activity in roots and leaves of Fe-deficient plants. Figure 3A (Additional file 1) shows that ICDH activity increased during the Fe deficiency treatment both in roots and in leaves. In particular, after 7 d of Fe starvation its increase was larger in roots, reaching a 2-fold increase, while in leaves this increase is around 100%. Northern blot analysis showed an over-expression of *ICDH* transcript both in roots and leaves during the progression of Fe deficiency. In particular, the expression of ICDH is larger after one day in leaves than in roots, while at 3 and 7 d the increased band intensity was similar in both tissues (Figure 3B).

### GS/GOGAT cycle under Fe deficiency

**Figure 4:**
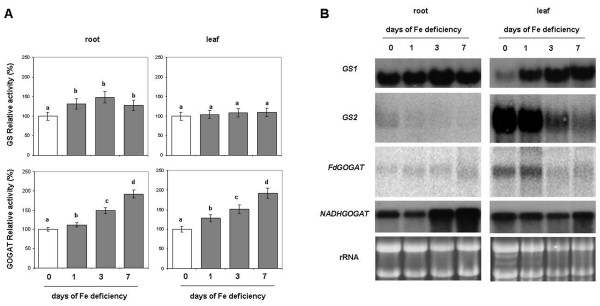
**(A) Enzymatic activity and (B) Northern Blot analysis of GS and GOGAT.** Assay and Northern Blot were performed on root and leaf of plant during the progression of Fe deficiency treatment. Sampling was performed at 0, 1, 3, 7 days after Fe withdraw. The meaning of the columns and the measurement units of enzymatic activities is as reported in Figure 2. Control (0 d) activity of GS was 259 and 520 nmol NADH mg^-1^ prot min^-1^, respectively for root and leaf. GS1, root isoform; GS2, leaf isoform. Control activity of GOGAT was 140 and 112 nmol NADH mg^-1^ prot min^-1^, respectively for root and leaf. Fd-GOGAT, ferredoxin-dependent isoform; NADH-GOGAT, NADH-dependent isoform (complete activity data are reported in Additional file 1). Data are means ± SE (n = 4). In the case of significant difference (P<0.05) values with different letters are statistically different.

As soon as NO_3_^-^ is reduced by NR and NiR, the NH_4_^+^ follows the N assimilation pathway through the GS/GOGAT cycle. Figure 4A and Additional file 1 (upper panels) shows the effect of Fe deficiency on the GS activity. This activity in root increased under Fe deficiency by about 30% in all days assayed, while it did not show any significant differences at the leaf level. Gene expression analysis of GS was performed on both the cytosolic (GS1) and plastidial (GS2) isoforms. As shown in Figure 4B (upper panels), GS1 was more expressed in the roots while GS2 was preferentially expressed in the leaves. In agreement with the increased enzymatic activity, the GS1 transcript was over expressed under Fe deficiency at the root level (particularly after 3 d), and in leaves at d 1, 3 and 7 (Figure 4B). The GS2 transcript was barely detected in roots while in leaves showed a decrease with the time (Figure 4B).

The GOGAT activity increased during Fe deficiency both at the root and at the leaf levels in all the assayed days and reaching its maximum (+90%) at 7 d (Figure 4A and Additional file 1 lower panels). Northern blot analysis was performed on the Fd-dependent and NAD(P)H-dependent GOGAT isoforms. Fd-GOGAT was the most abundant and more expressed in the leaves, while the NADH-GOGAT was similar in roots and leaves. At the root level, only the NAD(P)H-GOGAT transcript was clearly detected and showed an increase in its expression already after 3 d of Fe deficiency, accordingly with a major increase in its activity. In the leaves both isoform transcripts were detected, but while the NAD(P)H-GOGAT did not show any significant differences, the Fd-GOGAT showed a decrease in its expression with time. This last observation is in contrast with the increase in the GOGAT activity seen with the enzymatic assay (Figure 4B, lower panels). However, it is known that there may not be direct correlation between enzyme activity and transcript abundance.

### Amino acid concentration in Fe-deficient plants

**Figure 5:**
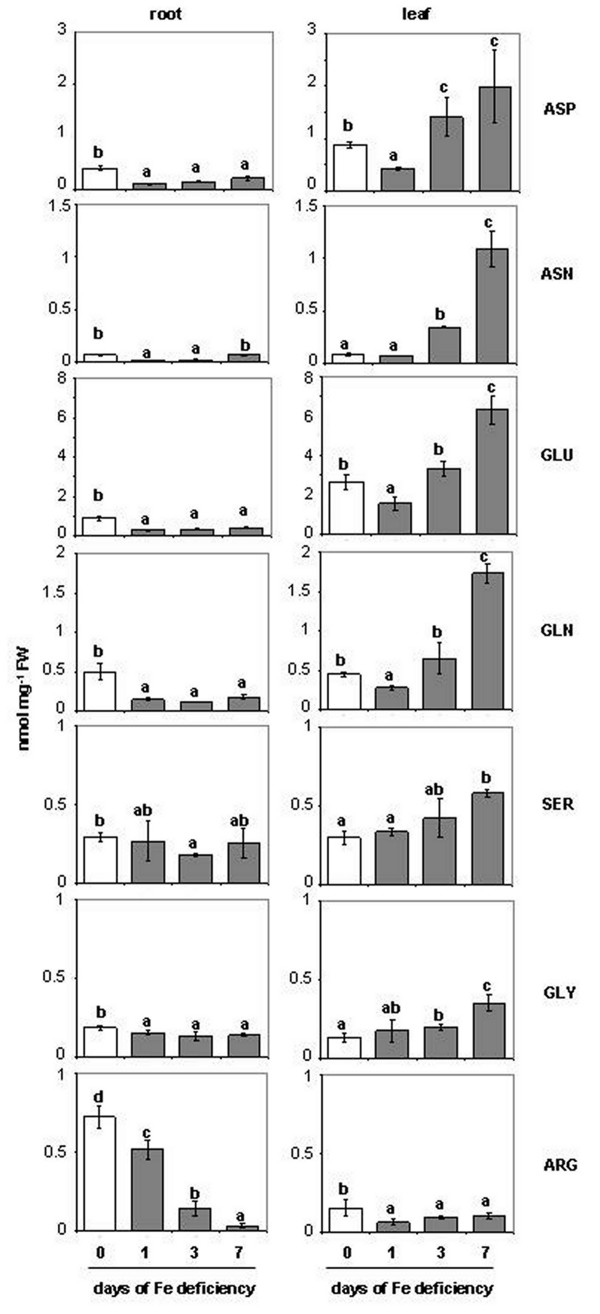
**Amino acid concentrations in root and leaf extracts of plants during the progression of Fe deficiency treatment.** Sampling was performed at 0, 1, 3, 7 days after Fe withdraw. The meaning of the columns is as reported in Figure 2. Abbreviation: Asp, aspartate; Asn, asparagine; Glu, glutamate; Gln, glutamine; Ser, serine; Gly, glycine; Arg, arginine (complete *aa* amounts data are reported in Additional file 2). Data are means ± SE (n = 4). In the case of significant difference (P<0.05) values with different letters are statistically different.

Figure 5 and Additional file 2 reports the amino acid (*aa*) concentration in roots and leaves of plants during the progression of Fe deficiency. The analysis of *aa* closely related to glycolysis and to the Krebs cycle, like, glutamate (Glu), glutamine (Gln), aspartate (Asp), asparagine (Asn), arginine (Arg), glycine (Gly), and serine (Ser) was chosen. In general, it is possible to note that *aa* concentration decreased in Fe-deficient roots, while it increased in leaves, as the Fe deficiency goes forward, with the sole exception of the Arg (Figure 5).

**Figure 6:**
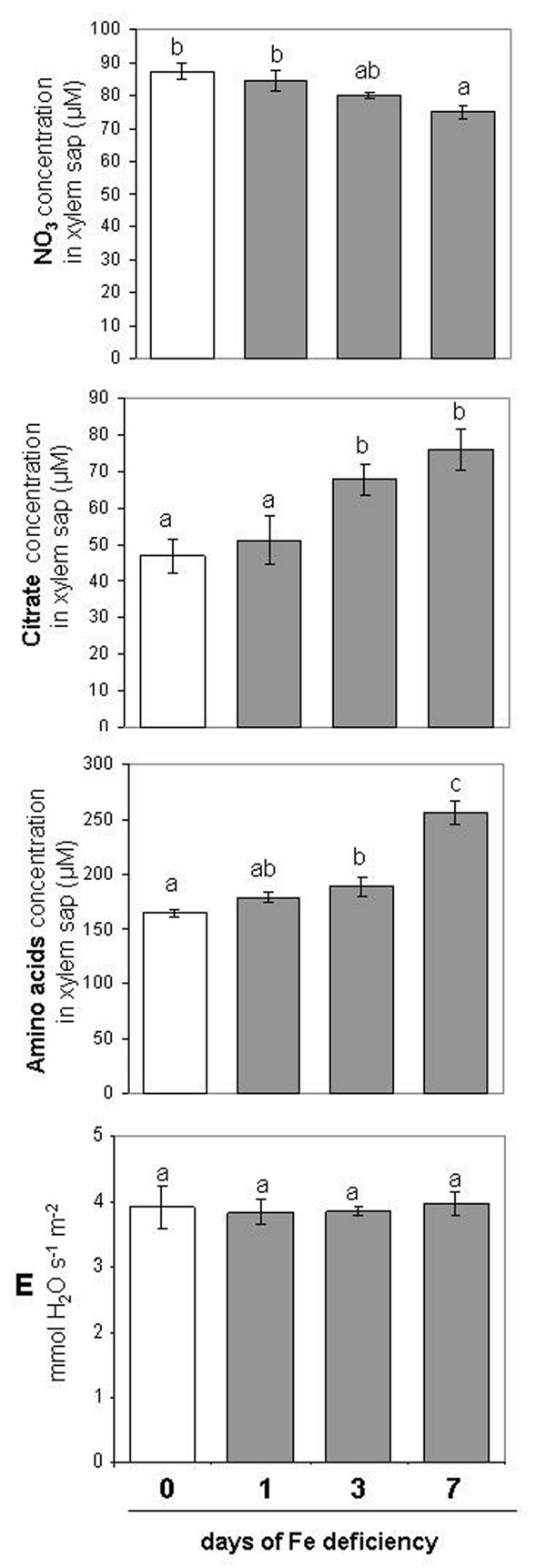
**Nitrate, citrate and total *aa *concentration in the xylem sap and plant transpiration rate (E).** Xylem sap was collected from plant during the progression of Fe deficiency treatment. Sampling was performed at 0, 1, 3, 7 days after Fe withdraw. The concentration is expressed as μM. Data are means ± SE (n = 3). In the case of significant difference (P<0.05) values with different letters are statistically different.

Table 1 reports for all the *aa* determined the differences between 0 d and 7 d. In particular, Asp showed a decrease in Fe-deficient roots (−49%) while it increased in Fe-deficient leaves (+125%). The Asn concentration differed at leaf level where the increase was about 13-fold at 7 d of -Fe condition compared to the control, while it did not show any difference in the roots (Figure 5). The change of Glu and Gln was very similar, but, in Fe-deficient tissues, Gln decreased more in root (−64%) and increased more in leaf (3-fold) when compared with Glu (−52% and +138%, in roots and leaves, respectively). Similarly, the concentration of Ser and Gly decreased in root while it increased in leaf, as Fe deficiency condition proceed. However, the Ser decrease in root was only significant at d 3 (−38%), while Gly decreased in root by about 25% after 7 d of Fe deficiency. At the same time both Ser and Gly increased in leaves (+94% and +160%, respectively). Interestingly, only the Arg showed a significant decreased concentration at d 7 in both roots (−96%) and leaves (−35%). The different *aa* concentration between roots and leaves prompted us to measure their translocation through the xylem. As shown in Figure 6 the concentration of total *aa* in xylem sap was increased in Fe-deficient roots and after 7d of Fe deficiency it reached the maximum level (+50%). At the same time also the citrate concentration was increased in the xylem sap at d 7 (+50%), confirming the data obtained by Abadía and co-workers [41]. On the contrary, at d 7 the concentration of NO_3_^-^ in the xylem was slightly decreased (−14%). The data collected from xylem sap are considered as an amount increase in nitrate, citrate and *aa*, since transpiration (E) rate did not change during the expression of Fe deficiency (Figure 6).

**Table 1:** Changes in amino acid concentration (%) in roots and leaves of cucumber plants

**Aa**	**+Fe (0d)/-Fe (7d)**		**N/C**	
	**root**	**leaf**		
Asp	−49	+125	C_4_H_7_NO_4_	0.25
Asn	0	+1.263	C_4_H_8_N_2_O_3_	0.50
Gln	−64	+350	C_5_H_10_N_2_O_3_	0.40
Glu	−53	+138	C_5_H_9_NO_4_	0.20
Gly	−25	+160	C_2_H_5_NO_2_	0.50
Ser	−12	+94	C_3_H_7_NO_3_	0.33
Arg	−96	−35	C_6_H_14_N_4_O_2_	0.67

Values are the ratio determined between 0 and 7 days of treatment. N/C is the ratio between Nitrogen and Carbon concentration in the molecule.

### ALT and AST activities under Fe deficiency

**Figure 7:**
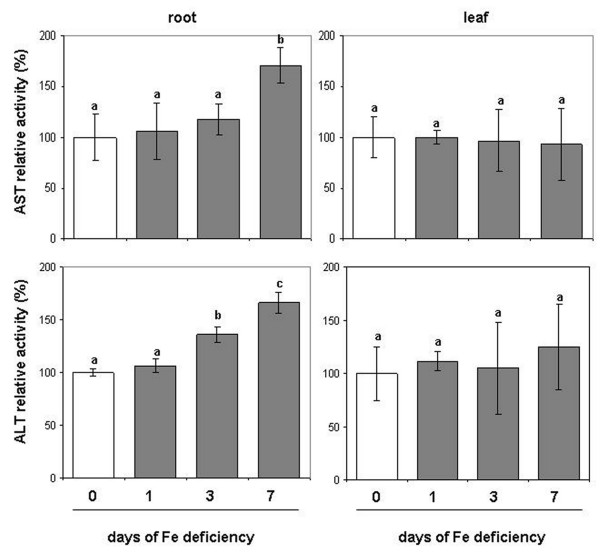
**Enzymatic assay of ALT and AST. The assay was performed on root and leaf of plant during the progression of Fe deficiency treatment.** Sampling was performed at 0, 1, 3, 7 days after Fe withdrawing. The meaning of the columns and the measurement units of enzymatic activities is as reported in Figure 2. AST activity of control (0 d) was 17 and 30 nmol NADH mg^-1^ prot min^-1^ for root and leaf, respectively. ALT activity of control (0 d) was 29 and 20 nmol NADH mg^-1^ prot min^-1^ for root and leaf, respectively (complete activity data are reported in Additional file 1). Data are means ± SE (n = 3). In the case of significant difference (P<0.05) values with different letters are statistically different.

We have assayed the activity of two enzymes that are recognised to be involved in the recycling of *aa* i.e. the alanine-aminotransferase (ALT) and the aspartate-aminotransferase (AST). AST was also considered, along with ICDH, as one of the main candidates in the synthesis of 2-OG [35]. As shown in Figure 7 (Additional file 1), during the progression of Fe deficiency the activity of these two enzymes increased only in roots, while it did not significantly change in leaves.

## Discussion

It is well documented that Fe deficiency strongly affects plant C metabolism altering all metabolic pathways directly or indirectly related with it [18,38,42]. Nitrogen assimilation represents one of the most important pathway related to C metabolism, but the mechanism(s) of its modulation under Fe deficiency is still not well known. Among the connecting points between C and N metabolism two seem to be important. The first is the PEPC activity and the second is the ICDH activity. The anaplerotic reaction catalysed by the PEPC to form the keto acid oxalacetate (OAA) is more likely essential for the replenishment of the TCA cycle intermediates, which are withdrawn for biosynthetic purposes, enabling net C skeleton synthesis [25]. Cytosolic ICDH uses the isocitrate to form 2-OG, which along with OAA, are considered essential substrates for N-assimilation and *aa* biosynthesis [37]. In agreement, we have previously demonstrated that the mitochondrial di- tricarboxylic acid carrier (DTC) was increased under Fe deficiency, suggesting an increased organic acid exchange between cytosol and mitochondria [38]. Furthermore, along with oxidative pentose phosphate pathway activity, ICDH activity produces reducing power (i.e. NADPH), useful for most of the metabolic changes occurring under this condition. In this work an increase in the cytosolic ICDH activity (Figure 3A) and ICDH transcript expression (Figure 3B) was observed in Fe-deficient roots. Also, the activity, the transcript and the amount of PEPC have been previously shown to increase in cucumber roots under Fe deficiency [43,44]. Moreover, these results suggested that there was an increase in the OAA and 2-OG production, and thereby an increase in the substrates necessary for the GS/GOGAT cycle, but also in the production of C skeletons for *aa* biosynthesis downstream the GS/GOGAT cycle activity.

In this work we have determined both the activity and the gene expression of GS and GOGAT at root and leaf levels. Under Fe deficiency, GS activity increased in roots whilst it did not show any substantial difference in leaves (Figure 4A). The increase in the GS1 activity was also supported by a proteomic study carried out in our lab on Fe-deficient cucumber roots [9]. Accordingly, the increase in the GS1 activity matched with a slight increase in the expression of its relative transcript, at least after 3 d of Fe deficiency (Figure 4A). Interestingly, at leaf level, the expression of the specific isoform GS2 decreased as compared with the control (d 0), while the root isoform GS1 was over-expressed also at the leaf level. For the leaves it is possible to hypothesise that the decrease in the expression of GS2 transcript may be in part compensated by the increase in the expression of GS1. If the amount of the expressed transcripts were considered altogether, no change in the total expression pattern would be found (Figure 4B). Concerning the GOGAT activity it increased in both roots and leaves during the Fe deficiency (Figure 4A). The increase in GOGAT activity was supported by an over-expression of its relative transcript only in roots where the increased expression of the NADH-dependent isoform was evident. In leaves a decrease in the expression of the Fd-dependent isoform was seen (Figure 4B) while the expression of the NADH-dependent isoform did not change.

If on one hand the Fe deficiency differentially affected the GS/GOGAT cycle in roots and leaves, on the other hand the upstream NO_3_^-^ reduction (mediated by NR) decreased in both tissues under the same condition (Figure 2). However, NR activity was less affected in roots than in leaves. It is worth to note that, while in leaves both the NR activity and its relative transcript were strongly diminished, particularly after 7 d of Fe deficiency induction. In the roots an opposite scenario occurred: the activity decreased by about 50%, while the transcript was up-regulated after 7 d of Fe deficiency (Figure 2B and C). Interestingly, the concentration of NR protein in roots showed a slight increase in the band intensity as the Fe deficiency proceeded (Additional file 3). These results suggest that the Fe-deficient root cells, in an effort to keep NO_3_^-^ reduction high, induced an over-expression of NR transcript and the relative protein but, the lack of Fe lowered the efficiency of the NR activity. This response seem to be rationale: among the metabolic factors modulating the gene expression of NR, NO_3_^-^ and *aa* concentration are very important. NO_3_^-^ positively regulates NR expression while, on the contrary, *aa* negatively regulate its expression [45]. The strong decrease in the NR expression at leaf level could be in agreement with the decrease in the NO_3_^-^ concentration and with the increase in the *aa* concentration. The opposite it is true at the root level, where the NO_3_^-^ concentration did not change, whilst the *aa* strongly decreased (see Figures 2A and 5). Moreover, the difference in the NR activity behaviour between roots and leaves could also reside both on the availability of ferredoxin (an Fe-S protein) as suggested by Alcaraz et al. [8] and in the compartmentalization of Fe. In fact, the chloroplast has been showed to contain more than 80% of the total Fe present in the leaves [46]. This might suggest a kind of hierarchy on the allocation of Fe between the cytosol and the chloroplast which will greatly penalise the activity of cytosolic Fe-dependent enzymes (i.e. NR), but not those in the stroma such as the GOGAT. In a condition of Fe deficiency this would mean to (i) sustain preferentially the photosynthetic electron transport chain (which contains several Fe atoms), (ii) decrease the competition in the use of reductants and, consequently, (iii) increase the capacity to fix and reduce CO_2_ in a condition in which the photosynthesis rate was diminished [47] and also observed in this work (Figure 1B). Moreover, low photosynthetic activity might limit NR activity through a decreased reductants availability [48]. The task to reduce nitrate should seem to be maintained in the roots which keep a NR activity greater than leaves and where the production of reductants was higher [19,49,50].

Taken together these results indicate that, in Fe-deficient roots the reduction of NO_3_^-^ decreased, the GS/GOGAT cycle and consequently the ammonia assimilation increased and the *aa* concentration largely decreased. In the Fe-deficient leaves, the NO_3_^-^ reduction was strongly decreased, the GS/GOGAT cycle slightly increased and the *aa* concentration strongly increased.

**Figure 8:**
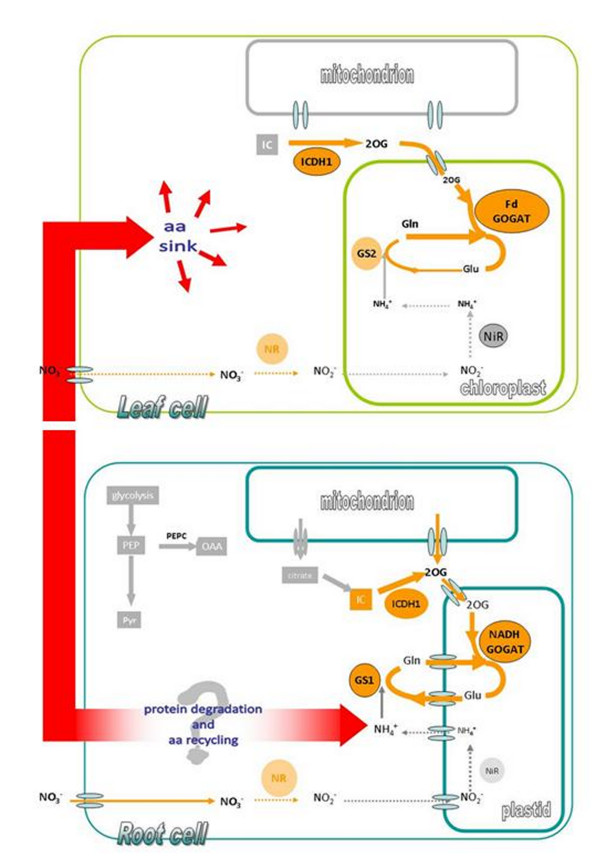
**Schematic representation of nitrogen metabolism changes occurring in Fe-deficient cucumber plants.** Orange arrows indicate the specific process investigate in this work, while the arrow thickness indicates an up- or down-activation of enzymatic activities. Red arrow indicates the possible recycle of protein and *aa* occurring under Fe deficiency in cucumber plants. Since NO_3_^-^ assimilation (NR activity) decreases and GS/GOGAT cycle increases in both root and leaf, the source of ammonia might come from a recycling of *aa* deriving from protein degradation [9]as suggested in ). The *aa* might be partially translocated to the leaf sustaining its metabolism as supported by data presented in this work (Figures 5 and 7).

The general decrease in *aa* content observed in Fe-deficient roots could be caused by different factors: (i) a major utilization of *aa* in the protein synthesis, (ii) an increase in their translocation to the leaves and (iii) a degradation and/or recycling of *aa*. Direct evidence supporting the first hypothesis have been documented in Fe-deficient cucumber root [51]. Concerning the second hypotheses, an increase in the total *aa* concentration in the xylem sap was observed during the progression of Fe deficiency (this work, Figure 6). Considering that the transpiration rate (E) determined did not change during this progression (Figure 6 and [52]) it would mean that the *aa* concentration in the xylem sap increased under Fe deficiency accordingly with the decrease of *aa* in roots. Concerning the last hypothesis it remains speculative, being supported only by indirect evidence. In this work it was found that the activity of two enzymes involved in the recycling of *aa*, AST and ALT, increased in roots but not in leaves (Figure 7). These data are also supported by proteomic studies conducted on cucumber [9] and *Medicago truncatula*[11] where the concentration of ALT and AST, respectively, were found to increase under Fe deficiency. Furthermore, a C-N hydrolase family protein has been identified in cucumber roots, and its concentration increased under Fe deficiency [9]. This family of enzymes is involved in N metabolism and catalyses the cleavage of the amino group from C skeletons (amino acid and protein) [53]. Hence, the amino groups released could be re-assimilated throughout the GS/GOGAT cycle into new *aa*. In Donnini et al. [9] an intriguing hypothesis, albeit speculative, was formulated: some proteins (e.g. actin, tubulin and globulin) might be used as a source of *aa*, carbon skeletons and N-NH_4_^+^ under Fe deficiency. We could hypothesise that in this condition a portion of the newly assimilated N might derive from proteins already present which are sacrificed for the survival of the plant. On the other hand, it appears that plants possess a regulated protein degradation machinery [54] and references therein that are particularly active during the stress response and senescence, leading to a continuous turnover of cellular proteins. As a consequence, the *aa* released in the roots could, in part, be transported to the leaves to keep N metabolism functioning (Figure 8). Indeed, the N assimilation process (i.e. NR plus GS/GOGAT) was affected more in leaves than in roots, and so the increase in *aa* concentration apparently depends mainly on their translocation from the roots.

## Conclusions

In conclusion, it is suggested that Fe deficiency strongly affected N metabolism by limiting NR activity and by increasing GS/GOGAT, in both root and leaf. Nevertheless under Fe deficiency root and leaf showed some important differences: the *aa* content in root decreased while in leaf it increased, and this changes might match the opposite NR gene expression. Indeed, NR showed an over-expression and a down-expression in root and leaf, respectively. Additionally, the different *aa* distribution in Fe-deficient plants might be due the a major translocation (together with other metabolites) via xylem from root to leaf. Thus, under Fe deficiency, root seems to respond more efficiently sustaining the whole plant by furnishing metabolites (i.e. *aa*, organic acids) to the leaves. A schematic representation of the features of the metabolic pathways is reported in Figure 8.

## Methods

### Plant growing conditions

Cucumber seeds (*Cucumi*s *sativus* L. cv Marketmore ‘76, F.lli Ingegnoli, Milano) were surface sterilized and sown in Agriperlite, watered with 0.1 mM CaSO_4_, allowed to germinate in the dark at 26°C for 3 d, and then transferred to a complete nutrient solution with 0.1 mM Fe(III)-EDTA. Growing conditions were as reported by Rabotti and Zocchi [55]. Thirty 7-day-old plants grown in the nutrient solution were transferred, after removal of cotyledons, to 10 L of the same solution without Fe. Sampling was performed after 0, 1, 3 and 7 days following induction of Fe deficiency and 4 h after onset of the photoperiod. For clarity, plants previously grown in the presence of Fe for 7 d and then transferred to a Fe-free solution are hereby referred to as 0 throughout the text.

Leaf gas exchange measurements were taken during 7 days of the experiment to characterize photosynthesis performance and gas exchange measurements with a portable photosynthesis system (CIRAS-2, PP System, USA). Measurements were carried on fully expanded, intact leaves of Fe sufficient and Fe deficient plants. Net CO_2_ assimilation rate and transpiration were assessed at a concentration of 330 μmol CO_2_, ambient relative humidity, 28°C chamber temperature and a photon flux density of 1500 μmol m-^2^ s^-1^. The instrument was stabilized according to manufacturer guidelines.

### RNA isolation, northern blot and semiquantitative RT-PCR analysis

RNA was extracted from root and leaf tissues according to Vigani et al. [56]. The gene-specific primers used to amplify the genes considered in this work are reported in Additional file 4. PCR was carried out on first-strand cDNA using *Taq* DNA polymerase (Promega) and the identity of the amplified fragments verified by sequencing. Fe-deficient and control tissues were pulverized in liquid nitrogen using a mortar and pestle and total RNA was extracted using Trizol Reagent (Invitrogen). Thirty micrograms of total RNA per lane were separated by electrophoresis at 5 V cm^2^ in a 1.3% (w/v) agarose gel containing 6% (v/v) formaldehyde, transferred to a Hybond-N1 nylon membrane (Amersham Bioscience) by capillary blotting in 20X SSC and then fixed by UV cross-linking. The blot was hybridized with a ^32^P-labeled cDNA probe for the entire coding sequence of all the genes considered in the study. Pre-hybridization and hybridization were conducted according to the nylon membrane manufacturer’s instructions. The membrane was washed for 10 min with 23 SSC in 0.1% (w/v) SDS at room temperature, with 13 SSC in 0.1% (w/v) SDS at 65°C for 20 min and then for 10 min with 0.13 SSC in 0.1% (w/v) SDS at 65°C.

The probes for the genes considered in this work were designed after an *in silico* analysis performed using the information available in the following databases:

BRENDA, http://www.brenda-enzymes.org;

NCBI, http://www.ncbi.nlm.nih.gov/;

TAIR, http://www.arabidopsis.org

We used PCR product fragments as cDNA probes obtained by amplification of the primers as reported in Additional file 4.

After a Blast analysis in the Cucurbit genome database CuGenDB ( http://www.icugi.org/cgi-bin/ICuGI/tool/blast.cgi), the probe sequences obtained displayed percentage identity values against Cucurbit genes as reported in Additional file 4. With regard to the GS2 probe, no specific information was available from the CuGenDB, but in GenBank NCBI showed 98% sequence identity with the GS2 sequence of *Cucumis melo* (accession n. AY773090).

Semiquantitative RT-PCR analysis and the analysis of Strategy I genes in cucumber plants were carried out according to Donnini et al. [9].

### Enzyme assay

Enzymes were extracted as described previously [49]. NADPH-dependent isocitrate dehydrogenase (ICDH) (EC 1.1.1.42) was assayed according to López-Millán et al. [42]. The NR (EC 1.7.1.1) assay was performed according to [57]. The GS (EC 6.3.1.2) assay was performed according to [58]. The NADH-GOGAT (EC 1.4.1.14) activity was assayed according to [59]. Alanine aminotransferase (EC 2.6.1.2) (ALT) activity was determined by using an assay kit (Cayman Chemical Company) following the manufacturer’s instructions, while aspartate aminotransferase (EC 2.6.1.1) (AST) activity was assayed according to [60].

### Western blot analysis

Western blot analysis was performed according to Vigani et al. [38]. The NR antibody (raised against spinach NR) was provided by Dr. Kaiser. The incubation with primary antibody, diluted in TBS-T buffer (Tris Buffered Saline, 0.1% Tween-20), was carried out for 2 h at room temperature. After rinsing with TBS-T buffer nitrocellulose membranes were incubated at room temperature for 2 h with a 1:10000 diluted secondary antibody (alkaline phosphatase-conjugated anti-rabbit IgG, Sigma). After rinsing in TBS-T (Tris Buffered Saline, 0.1% Tween-20) filters were incubated in 5-bromo-4-chloro-3-indolyl phosphate and nitroblue tetrazolium (FAST BCIP/NBT, Sigma) for the detection reaction.

### Amino acid analysis

Amino acid (*aa*) analysis was performed on 0, 1, 3, 7-d plants. Leaves and roots were harvested separately. Free *aa* were extracted from fresh tissues at 4°C, first in 80% ethanol over night, then in 60% ethanol for 1 h and finally in distilled water for 24 h. The supernatants of each sample were pooled, aliquoted and kept at −20°C. Free *aa* were determined by HPLC as described by Muller and Touraine [61].

### Collection and analysis of the xylem sap

Xylem sap was collected for 1 h after cutting the stem and 4 h after onset of the light period. The total xylem volume collected did not exhibit any significant difference among plants at any time sampled (data not shown). Nitrate concentration was determined according to [62]. Citrate concentration was determined according to [49]. Total *aa* concentration was determined according to [63].

### Protein determination

Total protein concentration was determined by the using dye-binding method of Bradford [64], using γ–globulin as a standard.

### Statistical analysis

All statistical analyses were conducted with Sigma-Stat® 3.1. Means were compared by Student’s *t* test at the P≤0.05 level in all cases.

## Abbreviations

*aa*: Amino acid; ALT: Alanine aminotransferase; Arg: Arginine; Asn: Asparagine; Asp: Aspartate; AST: Aspartate amonitransferase; G-3-P-DH: Glyceraldehyde 3-phosphate dehydrogenase; Glc-6P-DH: Glucose 6phosphate dehydrogenase; Gln: Glutamine; Glu: Glutamate; Gly: Glycine; GS: Glutamine synthetase; GOGAT: Glutamate synthase; ICDH: Isocitrate dehydrogenase; PEPC: Phospho*enol*pyruvate carboxylase; Ser: Serine.

## Competing interests

The authors declare that they have no competing interests.

## Authors’ contributions

AB carried out gene expression analysis, enzyme assays, amino acid analysis. GV carried out enzyme assays, collection and analysis of xylem sap, western blotting analysis, determination of physiological parameters and wrote the draft of the manuscript. GZ participated in the strategic planning of the work, data analysis and writing the final version of manuscript. All the authors contributed to the discussion of the results and took part to the critical revision of the manuscript. All authors read and approved the final manuscript.

## Supplementary Material

Additional file 1 **Enzyme activity expressed as as nmol NADPH mg**^**-1 **^**prot min**^**-1**^**.** Click here for file

Additional file 2 Concentration of amino acids. Click here for file

Additional file 3 **Western Blot analysis of nitrate reductase (NR) was performed on soluble fraction extracted from cucumber roots.** 0, 1, 3, 7 are the days after Fe withdrawing. Click here for file

Additional file 4 Oligonucleotide primers for RT-PCR. Click here for file
