# Supplementary material for: Iron deficiency affects nitrogen metabolism in cucumber (Cucumis sativus L.) plants
Source: BMC Plant Biol. 2012 Oct 11;12:189. doi: 10.1186/1471-2229-12-189 (PMC3539955; doi:10.1186/1471-2229-12-189)
Supplement: Additional file 1 — Enzyme activity expressed as as nmol NADPH mg-1 prot min-1. [file 1471-2229-12-189-S1.doc]

| **Table S2. Enzymatic activities assayed on both root and leaf tissues at 0,1, 3, 7 days of Fe deficiency. Data are expressed as nmol NADPH mg^-1^ prot min^-1^.** | | | | | |
| --- | --- | --- | --- | --- | --- |
| **Enzyme** | **tissue** | **days of Fe deficiency** | | | |
|  |  | **0** | **1** | **3** | **7** |
| NR | root | 5.6±0.3 | 3.6±0.2 | 3.6±0.2 | 2.9±0.1 |
|  | leaf | 9.8±0.5 | 5.1±0.3 | 3.4±0.2 | 2.0±0.1 |
| ICDH | root | 92.1±4.6 | 184.2±9.2 | 258.0±12.9 | 296.1±14.8 |
|  | leaf | 98.3±4.9 | 166.6±9.3 | 196.0±11.3 | 200.9±12.1 |
| GS | root | 259±12.9 | 339.3±16.9 | 235.3±11.8 | 331.5±16.6 |
|  | leaf | 520.2±26.1 | 541.3±27.1 | 564.2±28.2 | 569.4±29.8 |
| GOGAT | root | 140.1±7 | 156.9±7.8 | 209.3±10.5 | 268.8±13.4 |
|  | leaf | 112.3±5.6 | 143.3±7.2 | 169.7±8.4 | 244.9±12.3 |
| ALT | root | 29.4 ±1 | 32.3 ±2 | 41.7 ±3 | 50.3 ±5 |
|  | leaf | 20.3 ±5 | 22.3 ±2 | 21.7 ±9 | 29.5 ±10 |
| AST | root | 17.2 ±4 | 18.3 ±5 | 20.6 ±3 | 29.1 ±5 |
|  | leaf | 30.2 ±6 | 30.5 ±2 | 29.2 ±9 | 28.3 ±10 |
|  |  |  |  |  |  |
