# Supplementary material for: Iron deficiency affects nitrogen metabolism in cucumber (Cucumis sativus L.) plants
Source: BMC Plant Biol. 2012 Oct 11;12:189. doi: 10.1186/1471-2229-12-189 (PMC3539955; doi:10.1186/1471-2229-12-189)
Supplement: Additional file 2 — Concentration of amino acids. [file 1471-2229-12-189-S2.doc]

| **Table S3**. Amount of amino acids (nmol mg^-1^ FW^-1^) determined on +Fe (0) and +Fe/-Fe treated plants. Data are the means ±SD (n=4) | | | | | | | | |
| --- | --- | --- | --- | --- | --- | --- | --- | --- |
|  | **days of -Fe** | **ASP** | **ASN** | **GLU** | **GLN** | **SER** | **GLY** | **ARG** |
| root | 0 | 0.404 ± 0.036 | 0.064 ± 0.007 | 0.904 ± 0.107 | 0.495 ± 0.105 | 0.293 ± 0.032 | 0.186 ± 0.014 | 0.721 ± 0.070 |
|  | 1 | 0.106 ± 0.010 | 0.020 ± 0.002 | 0.328 ± 0.028 | 0.142 ± 0.016 | 0.266 ± 0.131 | 0.151 ± 0.016 | 0.514 ± 0.059 |
|  | 3 | 0.151 ± 0.021 | 0.023 ± 0.002 | 0.363 ± 0.039 | 0.111 ± 0.003 | 0.180 ± 0.012 | 0.131 ± 0.026 | 0.141 ± 0.047 |
|  | 7 | 0.207 ± 0.32 | 0.064 ± 0.007 | 0.428 ± 0.056 | 0.178 ± 0.024 | 0.257 ± 0.091 | 0.139 ± 0.012 | 0.030 ± 0.012 |
| leaf | 0 | 0.882 ± 0.053 | 0.078 ± 0.013 | 2.653 ± 0.395 | 0.442 ± 0.030 | 0.299 ± 0.044 | 0.135 ± 0.031 | 0.153 ± 0.048 |
|  | 1 | 0.420 ± 0.022 | 0.073 ± 0.001 | 3.288 ± 0.385 | 0.276 ± 0.031 | 0.338 ± 0.022 | 0.176 ± 0.074 | 0.063 ± 0.015 |
|  | 3 | 1.413 ± 0.370 | 0.340 ± 0.010 | 4.840 ± 0.934 | 0.648 ± 0.201 | 0.420 ± 0.123 | 0.201 ± 0.018 | 0.097 ± 0.006 |
|  | 7 | 1.990 ± 0.695 | 1.090 ± 0.169 | 6.306 ± 0.726 | 1.726 ± 0.127 | 0.579 ± 0.022 | 0.351 ± 0.053 | 0.100 ± 0.019 |
|  |  |  |  |  |  |  |  |  |
