# Supplementary figures and images for: Iron deficiency affects nitrogen metabolism in cucumber (Cucumis sativus L.) plants
Source: BMC Plant Biol. 2012 Oct 11;12:189. doi: 10.1186/1471-2229-12-189 (PMC3539955; doi:10.1186/1471-2229-12-189)

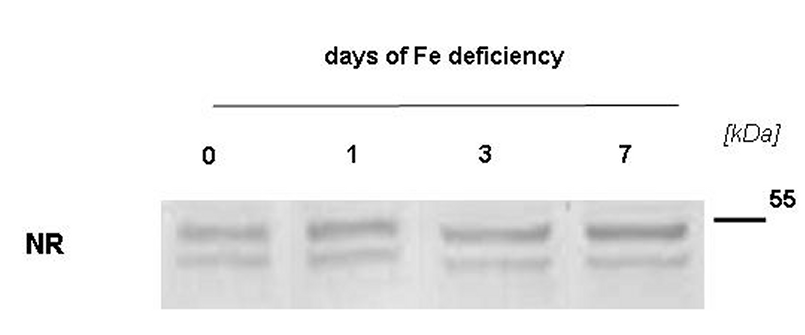

Supplement: Additional file 3 — Western Blot analysis of nitrate reductase (NR) was performed on soluble fraction extracted from cucumber roots. 0, 1, 3, 7 are the days after Fe withdrawing. [file 1471-2229-12-189-S3.tiff]
