# Supplementary material for: Iron deficiency affects nitrogen metabolism in cucumber (Cucumis sativus L.) plants
Source: BMC Plant Biol. 2012 Oct 11;12:189. doi: 10.1186/1471-2229-12-189 (PMC3539955; doi:10.1186/1471-2229-12-189)
Supplement: Additional file 4 — Oligonucleotide primers for RT-PCR. [file 1471-2229-12-189-S4.doc]

| **Table S1.** Primers designed for transcript identification and amplification | | | |
| --- | --- | --- | --- |
| oligonucleotide | | starting coding sequence | |
| primer name | sequence (5’-3’) | accession number^a^ | organisms |
| **ICDH1 for** | gggcatgcaagaactatgatg | AJ437269 | *Cucumis sativus* |
| **ICDH1 rev** | ctatgccttgaggagtctgg |
| **NR for** | cggattcaactggggagcggc | AY580989 | *Cucumis sativus* |
| **NR rev** | gtctacatgggatggcaagac |
| **FdGOGAT for** | ggatccgtacttgaaggcccaag | NM_129687 | *Arabidopsis thaliana* |
| **FdGOGAT rev** | cttgttactaagacgccaatgcc |
| **GS2 for** | ggatcccttccgtggtggcaac | NM_122954 | *Arabidopsis thaliana* |
| **GS2 rev** | gcttcgatcccaacactaggacc |
| **GS1 for** | tggatatsagaagcaaagccaggac | NM_125126 | *Arabidopsis thaliana* |
| **GS1 rev** | gtgagacgrgctcrttkccttcaccgtaagc |
| **NADHGOGAT_for** | tctgctgarccwcgtgaagtgca | NM_124725 | *Arabidopsis thaliana* |
| **NADHGOGAT_rev** | aaaggtgcwcagatwtttcaagctct |

^a^accession number related to GeneBak database NCBI (<http://www.ncbi.nlm.nih.gov/>)

Percentage values against the Cucurbit genes: ICDH probe showed a 99% of sequence identity with Csa021516 (annotation <http://www.icugi.org/cgi-bin/ICuGI/genome/search.cgi?gene=Csa021516&g=yes>), NR probe showed a 99% of sequence identity with Csa008224 (annotation <http://www.icugi.org/cgi-bin/ICuGI/genome/search.cgi?gene=Csa008224&g=yes>), Fd-GOGAT probe showed a 99% of sequence identity with Csa002676 (annotation <http://www.icugi.org/cgi-bin/ICuGI/genome/search.cgi?gene=Csa002676&g=yes>), NADH-GOGAT probe showed a 98% of sequence identity with Csa021126 (annotation <http://www.icugi.org/cgi-bin/ICuGI/genome/search.cgi?gene=Csa021126&g=yes>), GS1probe showed a 99% of sequence identity with Csa015274 (annotation <http://www.icugi.org/cgi-bin/ICuGI/genome/search.cgi?gene=Csa015274&g=yes>).
